# microRNA and Regulatory Networks of Human Macrophage During *Leishmania major* In Vitro Infection

**DOI:** 10.3390/ijms27198893

**Published:** 2026-10-07

**Authors:** Sara Habouria, Aymen Bali, Aurora Diotallevi, Ali Ben-Cheikh, Chiraz Atri, Mauro De Santi, Sara Maestrini, Luca Galluzzi, Dhafer Laouini, Fatma Zahra Guerfali

**Affiliations:** 1 Faculty of Sciences of Tunis, University Tunis El Manar, Tunis 2092, Tunisia; habouria.sara@gmail.com (S.H.); baliaymen@yahoo.fr (A.B.); 2 Laboratory of Transmission, Control and Immunobiology of Infections (LR16IPT02), Institut Pasteur de Tunis, Université de Tunis El Manar, Tunis 1002, Tunisia; alibencheikh87@gmail.com (A.B.-C.); chiraz_atri@yahoo.fr (C.A.); 3 Department of Biomolecular Sciences, University of Urbino Carlo Bo, 61032 Fano, Italy; aurora.diotallevi@uniurb.it (A.D.); mauro.desanti@uniurb.it (M.D.S.); sara.maestrini@uniurb.it (S.M.); luca.galluzzi@uniurb.it (L.G.)

**Keywords:** *Leishmania major*, microRNA, miR-21-5p, miR-146a-5p, macrophage, cutaneous leishmaniasis, host–pathogen interaction, gene silencing

## Abstract

*Leishmania* (*L.*) *major* modulates host microRNA (miR) expression to promote intracellular survival, yet the roles of specific miRs during early infection of human macrophages remain poorly defined. We investigated the dynamic regulation of miR-21-5p and miR-146a-5p in THP-1-derived macrophages infected with *L. major* over an early post-infection time course (3–48 h), alongside miR-30a-3p and miR-101-3p. Both miR-21-5p and miR-146a-5p were significantly upregulated during the early stages of infection (3–24 h) before declining by 48 h. Using antisense LNA inhibitors, we achieved independent single and dual silencing of both miRs, allowing us to dissect their functional contribution to host gene expression. In silico network analysis identified 887 shared and specific target genes, including NFKB1, MYC, STAT3, HIF1A, TNF, CXCL8, IL18, and XBP1. Silencing of miR-21-5p and miR-146a-5p at early time points relieved inhibitory control over several of these targets, notably increasing NF-κB, MYC, IL18 and XBP1 expression, and reducing TNF and sXBP1, consistent with their role as negative regulators of innate immune signaling. These findings identify miR-21-5p and miR-146a-5p as early, dynamic regulators of the human macrophage transcriptional response to *L. major* infection and identify their target genes as candidate biomarkers and therapeutic targets in cutaneous leishmaniasis.

## 1. Introduction

Leishmaniasis is a vector-borne disease caused by *Leishmania* (L.) parasites, affecting various host species, including humans. It manifests in three principal clinical forms: cutaneous, mucocutaneous, and visceral, with symptoms ranging from localized skin or mucosal ulcers that can be self-healing to severe systemic involvement that mainly affects the liver and spleen [1]. These diverse clinical manifestations are determined by the interplay between the host immune response and the infecting *Leishmania* species. Among the different parasite species, *L. major* is one of the principal causes of cutaneous leishmaniasis worldwide [2,3]. Macrophages, as innate immune cells, play a crucial role in defending the host against pathogens. However, they are also the principal host cells in which the parasite survives and replicates [4]. Therefore, *Leishmania*, as an obligatory intracellular parasite, has evolved mechanisms to evade the host’s immune response. These include escaping the complement system by expressing surface molecules such as lipophosphoglycan (LPG) and metalloprotease GP63 [5,6] and inhibiting antigen processing and presentation [7]. Once inside the macrophage, the parasite targets essential cellular mechanisms, particularly host-derived iron acquisition, for survival and pathogenicity [8] and it controls cell signaling pathways and affects the production of pro-inflammatory cytokines necessary for intracellular pathogen clearance [9,10]. One of the potential mediators of this host–parasite interaction is the modulation of host microRNA (miR) expression by the parasite. MiRs are small non-coding RNA molecules of approximately 22 nucleotides in length. This single-stranded RNA mainly regulates gene expression at the post-transcriptional level, thereby leading to either mRNA degradation or translational repression [11]. A single miRNA can potentially regulate a wide array of target mRNAs because its short sequence allows flexible base pairing with multiple targets. Binding does not require perfect complementarity, particularly outside the seed region, enabling a single miRNA to interact with numerous mRNAs through partial pairing within their 3′ untranslated regions (3′UTRs). This molecular versatility enables miRs to coordinate the regulation of multiple signaling pathways simultaneously. Through this combinatorial regulation, miRs fine-tune gene expression networks that control essential biological processes, such as immune activation, inflammation, and host defense against pathogens. Within this context, they are involved in the modulation of macrophage biology [12], including the control of their activation dynamics (M1 versus M2 polarization) and cytokine secretion profiles. In recent years, dysregulated miR expression has attracted heightened attention, as miRs act as regulators and biomarkers of immune responses across a wide range of diseases. However, their specific functions in controlling macrophage gene expression during cutaneous leishmaniasis remain largely uncharacterized. Our previous comprehensive screening of 365 miRs during *L. major* infection of Monocyte-Derived Macrophages (MDM) from PBMC revealed that approximately 20% of them displayed distinct patterns of expression [13,14]. Emerging evidence indicates that different *Leishmania* parasite species actively modulate host miRNA expression. Comprehensive analysis of the literature revealed several miRNAs that play pivotal regulatory roles in immune pathways activated during leishmaniasis. Based on all these insights, we selected four miRNAs: miR-21 [15], miR-30a [13], miR-101 [16], and miR-146a [17,18] for deeper investigation. These four miRs were chosen not only for their established roles in regulating immune and inflammatory processes in different major and neglected human tropical diseases [17,19], but also for their potential to reveal novel post-transcriptional mechanisms that govern macrophage responses during cutaneous leishmaniasis.

In this study, we aimed to investigate the dynamic regulation of miR-21-5p and miR-146a-5p across early post-infection kinetics and to determine their functional role in the human macrophage transcriptional response during *L. major* infection. These miRs were silenced individually and in combination, and integrated in silico target prediction was performed to identify key immunomodulatory genes. The study further aimed to identify miR-21-5p and miR-146a-5p target genes as candidate biomarkers and therapeutic targets in cutaneous leishmaniasis.

## 2. Results

### 2.1. Evaluation of the In Vitro Infection

**Figure 1:**
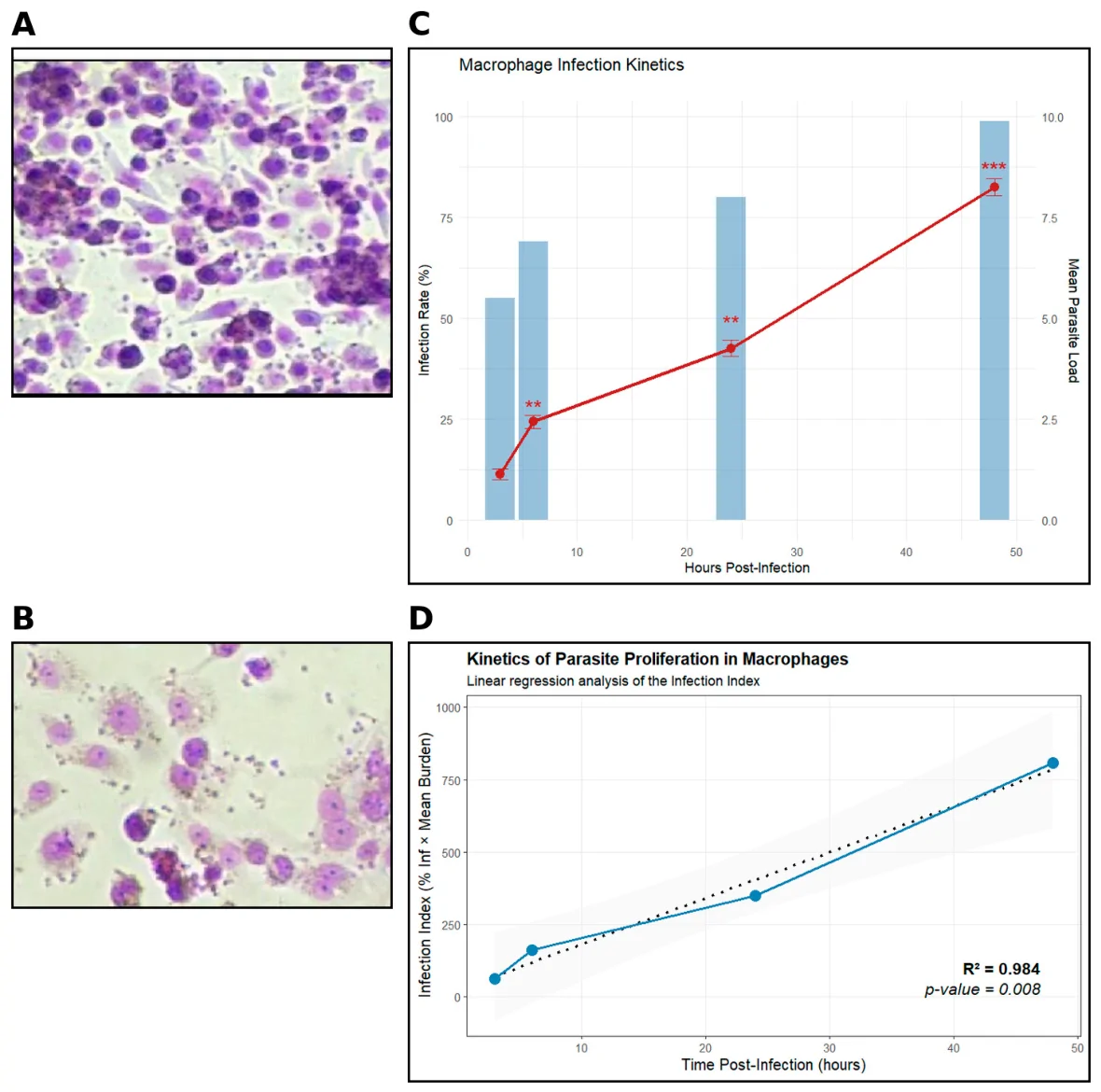
In vitro infection kinetics of human macrophage cells by *L. major*. MGG-stained slides at 24 h (**A**) and 48 h (**B**) revealed the presence of amastigotes within the macrophages, confirming successful establishment of the infection in this in vitro model. (**C**) At 3 h post-infection, 55% of macrophages were infected. At 6 h, 69% of cells were infected, with 2.32 parasites per cell. At 24 h, 80% of cells were infected, with an average of 4.38 parasites per cell. At 48 h, 99% of cells were infected, with an average of 8.16 parasites per cell. (**D**) Infection index (calculated as % infection × mean parasite burden). The dashed line represents the linear regression fit (R^2^ = 0.984), shown here as a descriptive trend indicator across the four time points rather than an assumption of strictly linear infection kinetics. *p* values < 0.01 (**), or <0.001 (***) were considered statistically significant.

Human THP-1 monocytes were differentiated into macrophage-like cells and infected in triplicate with PNA− purified metacyclic promastigotes of *L. major* MHOM/TN/94/GLC 94 at a parasite-to-cell ratio of 10:1. Infection was evaluated at 3, 6, 24, and 48 h post-infection. Non-infected cells maintained in complete medium and LPS-stimulated cells were included as negative and positive controls, respectively. To assess the success of this in vitro infection model, we performed MGG staining and examined the macrophages under a bright-field microscope. MGG-stained NI cells showed well-differentiated macrophages with a typical morphology, good adherence, and sustained survival throughout the observation period, with no intracellular parasites detected. These control cells provided a reference for evaluating the morphological status of the macrophages and confirming the absence of infection in the NI condition. The stained IF slides clearly revealed the presence of amastigotes within the macrophages, confirming the progressive establishment of infection (Figure 1A,B). In order to quantify the infection rate and the parasite load at various time points post-infection (3 h, 6 h, 24 h, and 48 h), at least 100 MGG-stained cells were randomly selected for each condition. The results show that both the infection percentage and the parasitic load within each infected macrophage increased significantly over time (Figure 1C). Even at the earliest time point, 3 h post-infection, 55% of macrophages were infected, with an average of 1.11 ± 0.12 parasites per cell. The infection rate increased to 69% of cells at 6 h post-infection, and the parasitic load nearly doubled, to 2.32 ± 0.18 parasites per cell. As the infection progressed to 24 h, a substantial rise in both the infection rate and the parasite load was observed: approximately 80% of macrophages were infected at this time point, each hosting an average of 4.38 ± 0.24 parasites. Finally, at 48 h post-infection, nearly all macrophages (99%) displayed signs of infection, with an average of 8.16 ± 0.25 parasites per cell. To assess the total parasite load, an Infection Index was calculated (Figure 1D). We observed a significant time-dependent increase in the Index (*p* < 0.01). This cumulative metric confirms sustained intracellular parasite persistence within the infected macrophages over the observation period.

### 2.2. Evidence-Based miRNA Candidate Selection

Through an integrative approach combining evidence-based literature curation with our laboratory’s primary screening data, we identified a high-confidence panel of four miRNAs: miR-21-5p, miR-146a-5p, miR-30a-3p, and miR-101-3p. These miRs are characterized by their significant regulatory potential within the human macrophage-pathogen interactome during *L. major* infection.

### 2.3. Housekeeping Gene for miR Normalization

**Figure 2:**
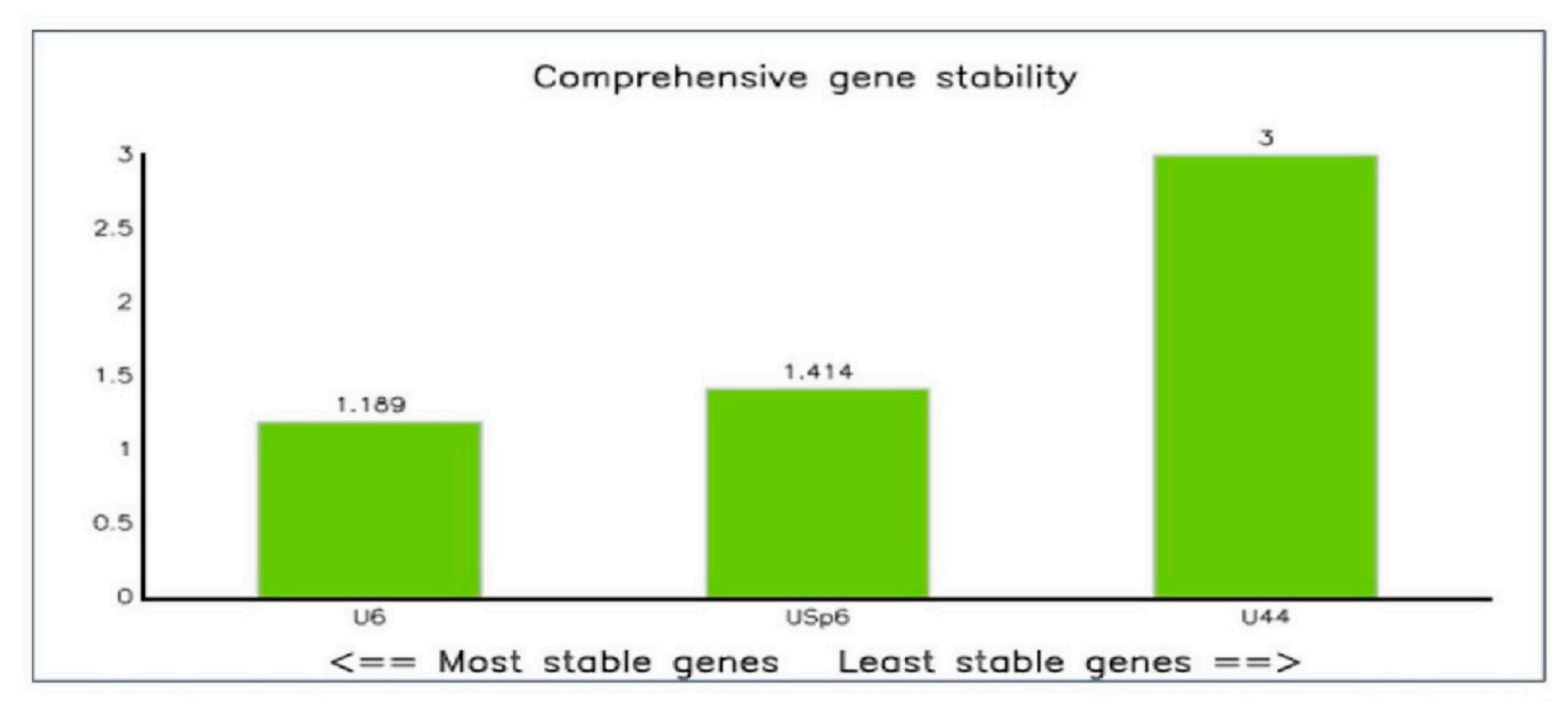
Stability ranking of miR housekeeping genes by RefFinder. The stability of each candidate reference gene for miR normalization (SNORD6 (U6), SNORD44 (U44), and the exogenous RNA spike-in UniSp6) was evaluated using the online tool RefFinder (combining BestKeeper, comparative ΔCt, NormFinder, and GeNorm). U6 exhibited the highest stability among the genes tested.

To identify the most suitable housekeeping gene for normalizing miR expression, the stability of three candidate genes—U6 and U44 as endogenous genes and RNA spike-in UniSp6 as an exogenous gene—was evaluated by the online-based tool RefFinder. Four different algorithms were combined to generate the ranking order (better–good–average). Our results demonstrated that U6 exhibited the highest stability among the genes tested (Figure 2). It ranked as the top-performing reference gene across most of the algorithms used, indicating its reliability for normalizing miR expression. UniSp6, the exogenous gene, showed the second-best stability, followed by U44. This ranking order was consistent with the findings obtained from both the NormFinder and the delta Ct methods. In the GeNorm analysis, both U6 and UniSp6 occupied the top rank, while UniSp6 outperformed U6 in the BestKeeper algorithm. Notably, U44 consistently ranked lowest across all the programs evaluated. Based on these results, we selected U6 as the endogenous housekeeping gene and UniSp6 as the exogenous housekeeping gene for normalizing miR expression. Moreover, UniSp6 serves as a plate calibrator across different runs, ensuring the consistency and comparability of the RT and qPCR steps across all samples. In particular, UniSp6 was uniformly detected in all samples from different runs, indicating that our experimental procedures were performed under consistent conditions and generated high-quality, comparable outcomes.

### 2.4. miR Expression

The expression profiles of miR-21-5p, miR-146a-5p, miR-30a-3p and miR-101-3p showed dynamic differential expression in IF compared to control cells NI and between IF themselves across different post-infection time points.

**Figure 3:**
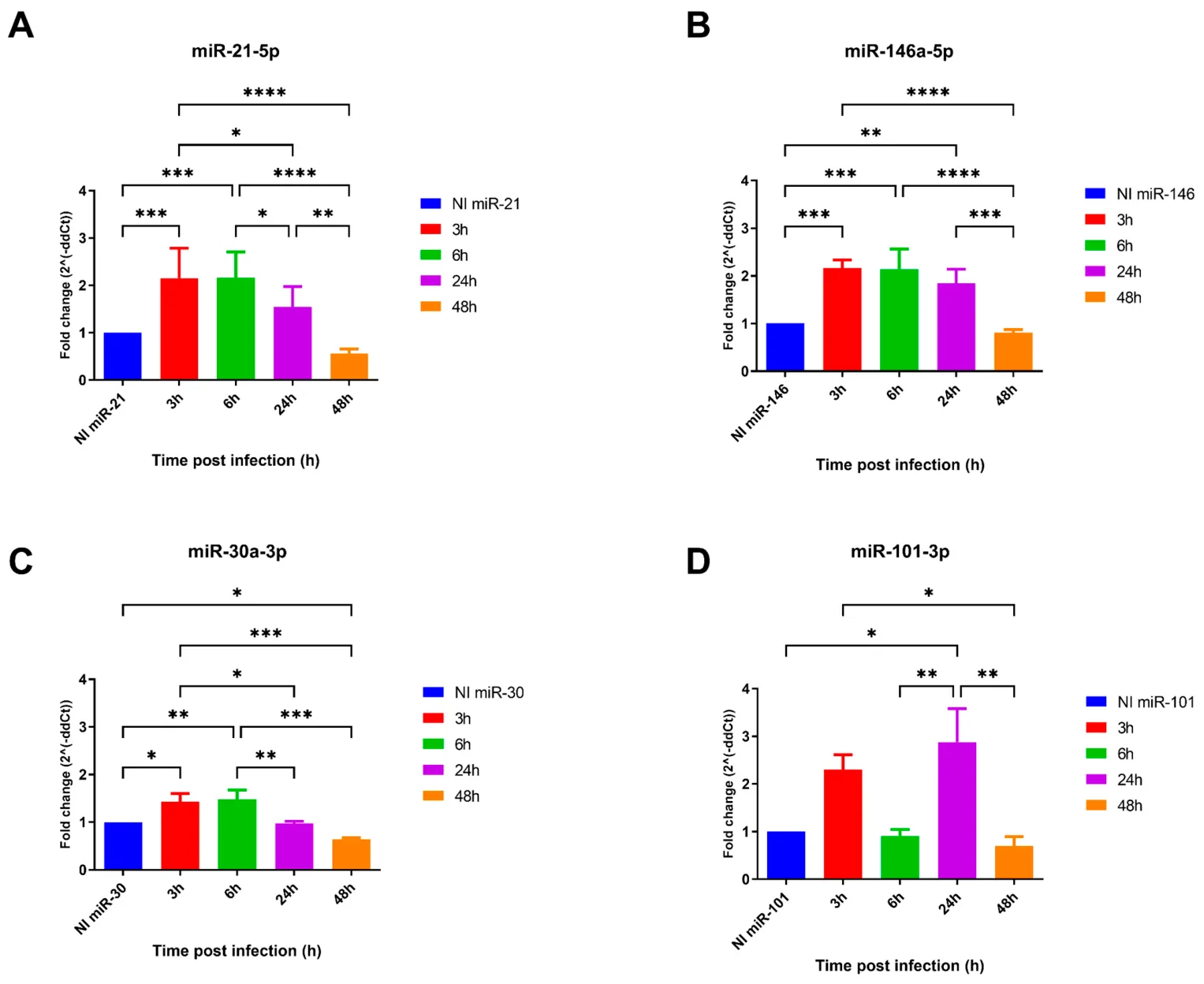
Differential expression of miR over time post-infection. Dynamic expression pattern of miR-21-5p (**A**), miR-146a-5p (**B**), miR-30a-3p (**C**), and miR-101-3p (**D**) in macrophages during the infection process (IF), showing significant upregulation from the early stage of infection. *p* values < 0.05 (*), <0.01 (**), <0.001 (***), or <0.0001 (****) were considered statistically significant.

Our findings demonstrated a significant upregulation of miR-21-5p in IF compared to NI from the earliest stages of infection and up to 24 h (Figure 3A). This upregulation was highly significant at 3 h (*p* < 0.001) and 6 h (*p* < 0.001). However, from 24 h onward, miR-21-5p expression started to decrease progressively and showed a significant reduction compared with the earlier time points 3 h (*p* < 0.05) and 6 h (*p* < 0.05). By 48 h, miR-21-5p was significantly downregulated in comparison with the earlier infection time points 3 h (*p* < 0.001), 6 h (*p* < 0.001), and 24 h (*p* < 0.01). miR-146a-5p showed a significant upregulation during early infection compared with NI cells (Figure 3B). This upregulation started at 3 h (*p* < 0.001) and was maintained at 6 h (*p* < 0.01), and 24 h (*p* < 0.05) post-infection. However, by 48 h post-infection, miR-146a-5p expression was significantly downregulated relative to IF cells at the earlier time points: 3 h (*p* < 0.001), 6 h (*p* < 0.01), and 24 h (*p* < 0.01) post-infection. Notably, no significant difference in miR-146a-5p expression was observed between 48 h IF cells and NI cells. MiR-30a-3p was significantly upregulated at 3 h (*p* < 0.05) and 6 h (*p* < 0.01) compared to NI controls (Figure 3C). However, its expression level decreased significantly by 24 h relative to the earlier time points, 3 h (*p* < 0.05) and 6 h (*p* < 0.01), and decreased further at 48 h compared with 3 h (*p* < 0.001) and 6 h (*p* < 0.001). Notably, at 48 h, miR-30a-3p was significantly downregulated in IF compared with the NI control (*p* < 0.05). The miR-101-3p profile displayed a distinctive biphasic expression pattern, unlike the other miRs analyzed, characterized by pronounced fluctuation in IF across the infection time course (Figure 3D). The expression of miR-101-3p was significantly upregulated at 3 h (*p* < 0.05) relative to the NI control, followed by a marked decrease at 6 h. Interestingly, the expression level rebounded at 24 h, showing a significant upregulation compared with the NI control (*p* < 0.05) and to 6 h (*p* < 0.01). By 48 h, miR-101-3p expression decreased significantly relative to 24 h (*p* < 0.01).

### 2.5. Prediction of miR’s Target Genes

**Figure 4:**
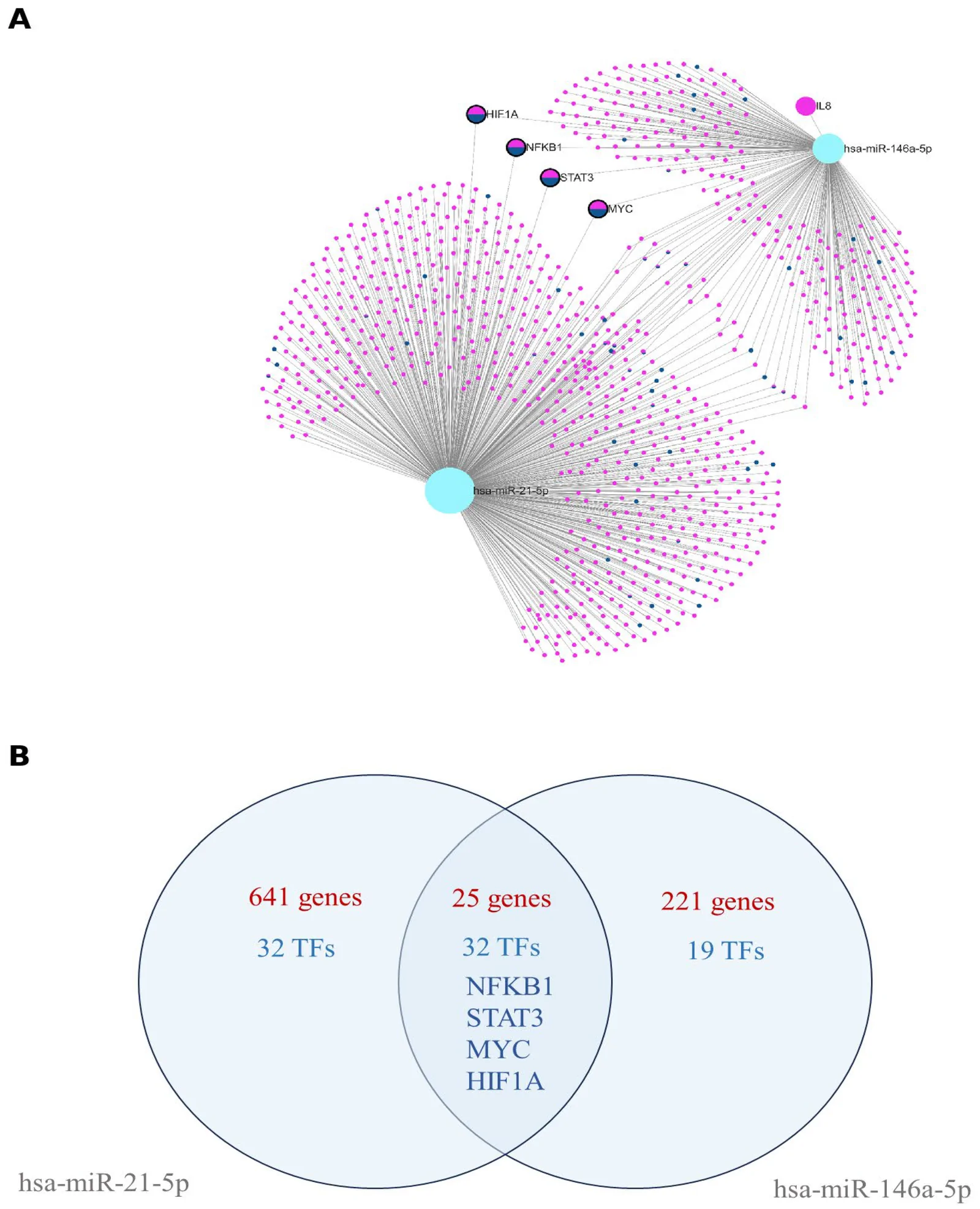
In silico regulatory network of hsa-miR-21-5p and hsa-miR-146a-5p. Computational network analysis of hsa-miR-21-5p and hsa-miR-146a-5p (large light-blue hub nodes) reveals a complex regulatory interactome. The integrated network comprises 887 target genes and 61 transcription factors interconnected by 963 edges, with multiple shared targets including the transcription factors HIF1A, NFKB1, STAT3, and MYC (**A**). Simplified Venn diagram summarizing the regulatory overlap between hsa-miR-21-5p and hsa-miR-146a-5p, showing their respective target genes and transcription factors, as well as shared target genes and the common transcription factors HIF1A, NFKB1, STAT3, and MYC (**B**).

In silico analysis of miR-21-5p and miR-146a-5p identified a complex regulatory network comprising 887 target genes and 61 transcription factors interconnected by 963 edges (Figure 4). Functional enrichment analysis using the KEGG database revealed 55 significant pathways (FDR < 0.05) (Supplementary Table S1). We focused on pathways closely associated with the innate immune response during infection, including the Toll-like receptor pathway, TGF-beta signaling pathway, apoptosis, cell cycle, leishmaniasis, cytokine–cytokine receptor interaction, JAK–STAT signaling pathway, and Chemokine signaling pathways, as those directly implicated in leishmaniasis pathology. A set of target genes was selected for downstream analysis. This selection prioritized genes co-regulated by both miRNAs and represented in at least two of these pathways, namely *NFKB1*, *MYC*, and *STAT3*. In addition, we included key immunological effectors previously reported in the literature in the context of leishmaniasis pathogenesis, specifically HIF1A, TNF, CXCL8, IL18, and XBP1, along with its active spliced isoform, sXBP1.

### 2.6. Selection of miR-21-5p and miR-146a-5p for Functional Silencing

For the remainder of this study, we selected miR-21-5p and miR-146a-5p for further investigation, given their marked and sustained upregulation during the early stage of infection. Both were targeted for silencing to assess their contribution to infection persistence, and we then examined the predicted target genes mediated by these miRs.

**Figure 5:**
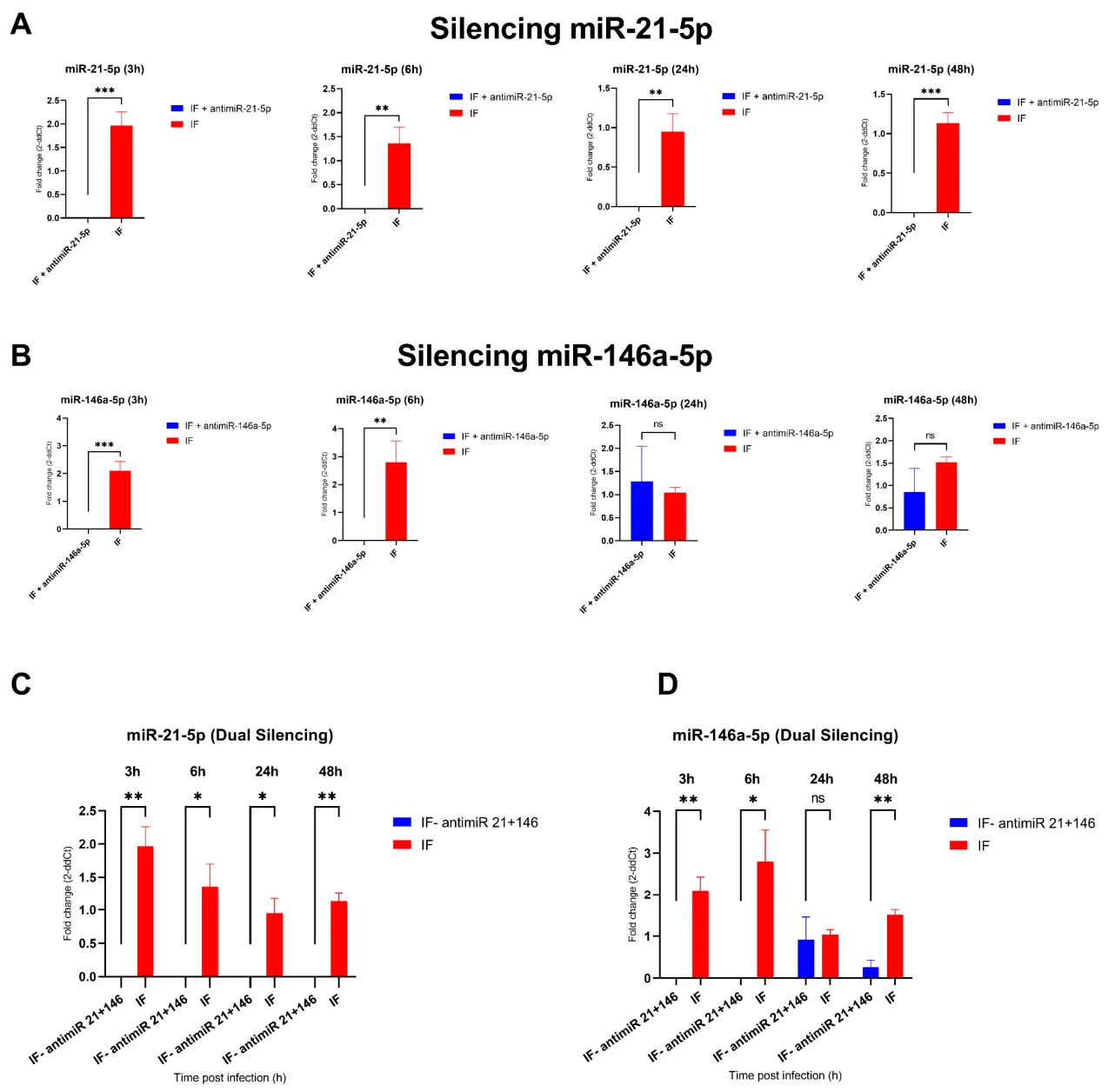
Silencing efficiency of miR-21-5p and miR-146a-5p, individually and in combination, over time post-infection. Anti-miR-21-5p treatment effectively silenced miR-21-5p (**A**) in infected cells (IF) at 3, 6, 24, and 48 h post-infection. Anti-miR-146a-5p treatment effectively silenced miR-146a-5p (**B**) in infected cells (IF) at 3 and 6 h post-infection; silencing was still observed at 48 h, although the effect was less pronounced at this later time point. Dual anti-miR-21-5p + 146a-5p co-transfection effectively silenced miR-21-5p (**C**) and miR-146a-5p (**D**) in infected cells (IF) across the same time course, following the same pattern as single-miR silencing. *p* values < 0.05 (*), <0.01 (**), or <0.001 (***) were considered statistically significant. ns: non significant.

MiR-21-5p silencing efficiency:

Transfection with anti-miR-21-5p resulted in robust and complete silencing of endogenous miR-21-5p expression, achieving 100% inhibition relative to infected cells across all measured time points (with no detectable qPCR amplification signal at the measured time points). This strong validation confirms the effectiveness and stability of the silencing, providing a reliable approach for investigating the functional role of miR-21-5p and analyzing the expression of its target genes (Figure 5A).

miR146a-5p silencing efficiency:

At early time points, anti-miR-146a achieved complete, 100% inhibition of miR-146a-5p expression, with no detectable qPCR amplification signal. This provides very strong validation for interpreting target gene data at 3 h and 6 h. Although silencing persisted at 24 h and 48 h, its efficacy decreased over time (Figure 5B).

**Figure 6:**
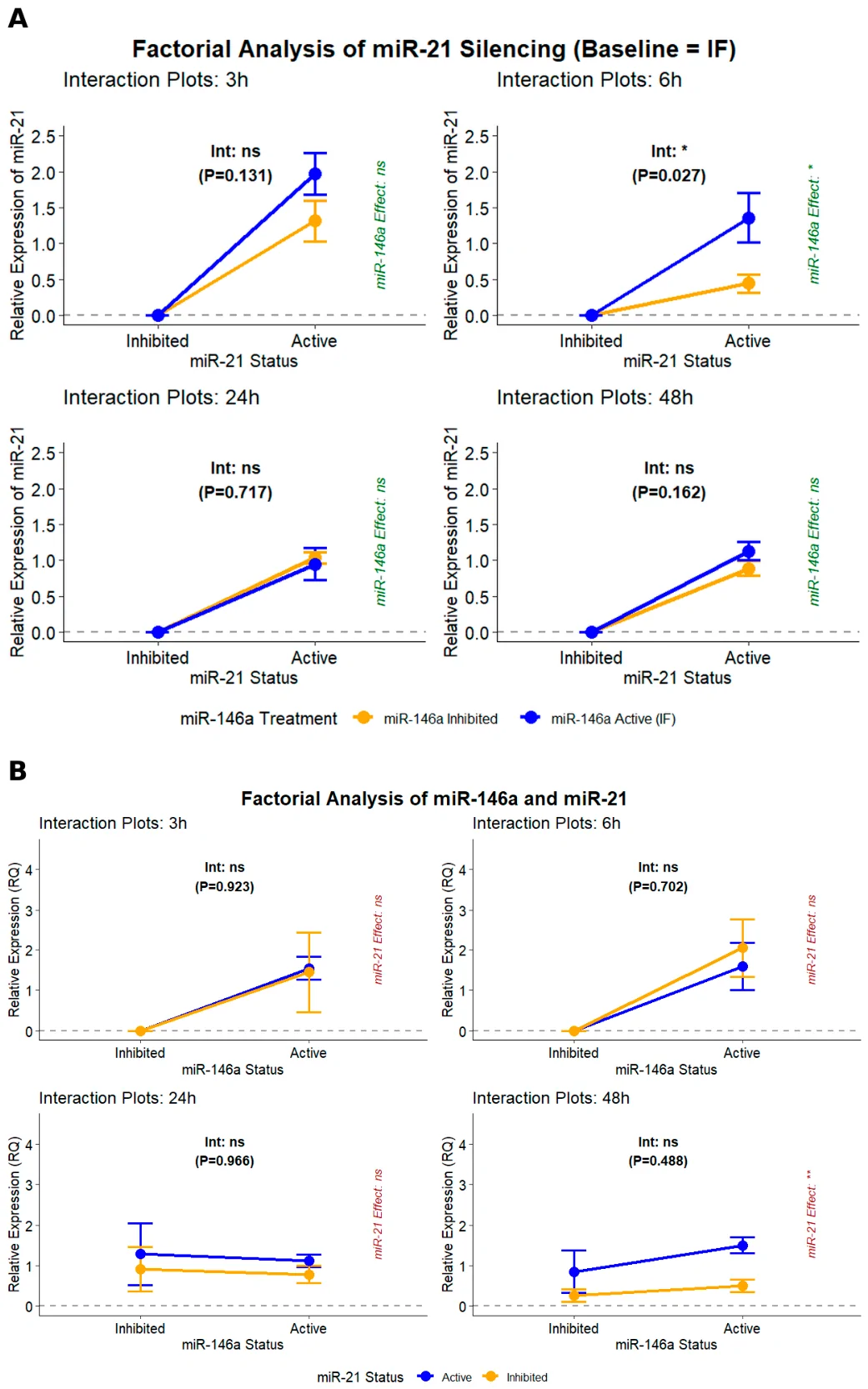
Factorial analysis of dual silencing of miR-21-5p and miR-146a-5p over time post-infection. Interaction plots showing that co-transfection of anti-miR-21-5p and anti-miR-146a-5p did not compromise the silencing efficacy of either compound at any time point, confirming the technical feasibility and compatibility of the dual-silencing approach used for the downstream functional analyses. int: interaction for dual siencing. miR-146a effect (panel **A**) and miR-21 effect (panel **B**) correspond to the inhibition effect of each micro-RNA. *p* value < 0.05 (*), or <0.01 (**) is considered statistically significant. ns: non significant.

Dual antimiR silencing efficiency:

In the dual anti-miR silencing setup, the results demonstrate that co-transfection of anti-miR-21 and anti-miR-146a did not compromise the silencing efficacy of either compound across all time points (Figure 5C,D and Figure 6). This confirms the technical feasibility and compatibility of the approach, validating simultaneous inhibition for the functional analyses that follow.

### 2.7. Target Genes Analysis

From the previously selected pathways, we identified a panel of genes as potential targets of miR-21-5p, miR-146a-5p, or both. To evaluate the regulatory impact of these miRNAs, we investigated the expression levels of these targets following miR silencing. The functional analysis focused on early time points post-infection, as our validation assays confirmed 100% silencing efficiency for both miRNAs (i.e., no detectable qPCR amplification signal).

**Figure 7:**
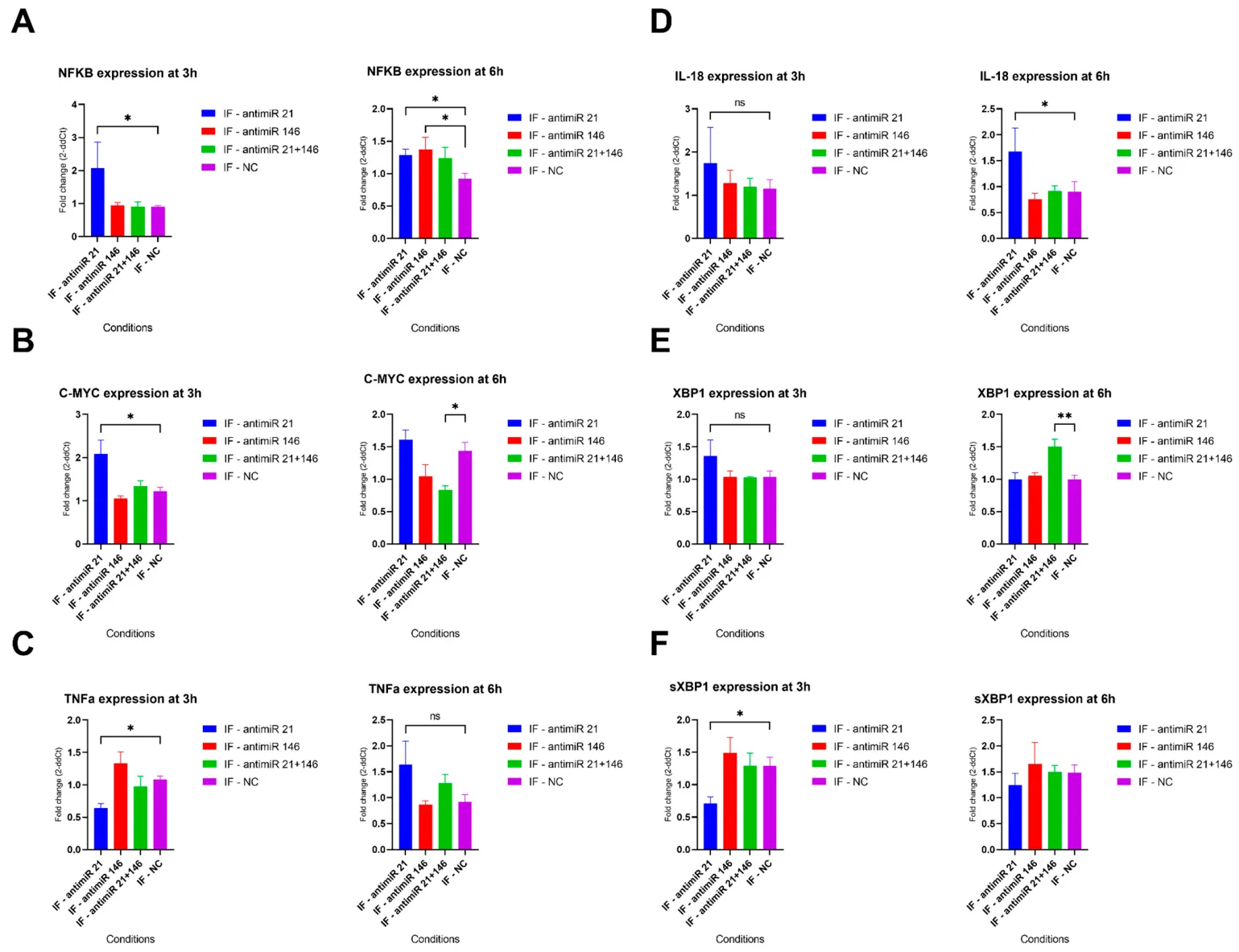
Expression of NFKB1, MYC, TNF, IL18, and XBP1 (and its spliced form sXBP1) under different conditions following miR silencing. (**A**) At 3 h, miR-21-5p silencing resulted in upregulation of NFKB1 expression by +67.4% compared with the infected control (IF-NC); this effect remained significant, though reduced (+36.2%), at 6 h. miR-146a-5p silencing significantly increased NFKB1 expression by +51.1% at 6 h. (**B**) At 3 h post-infection, inhibition of miR-21-5p produced a +77.3% increase in MYC expression relative to the control; by 6 h, the regulatory pattern reversed, resulting in a decrease of −41.3% in this gene’s expression level. (**C**) miR-21-5p silencing significantly reduced TNFα expression by −39.2% compared with the scrambled control (IF-NC); miR-146a-5p inhibition and the combined inhibition had no significant effect on TNF regulation. (**D**) Anti-miR-21-5p significantly increased IL18 expression relative to the negative control, reaching +97.5% at 6 h; miR-146a-5p inhibition produced a slight decrease in IL18 expression over time, and no significant changes were observed after dual inhibition. (**E**) Dual silencing of miR-21-5p and miR-146a-5p at 6 h led to a significant increase in XBP1 expression (+57.1%), exceeding the effect of either inhibitor alone. (**F**) At 3 h, miR-21-5p inhibition significantly downregulated sXBP1 expression by −43.8% relative to the scrambled negative control (IF-NC). *p* values < 0.05 (*) or <0.01 (**) were considered statistically significant. ns: non significant.

NFKB1 regulation: Among all the treatments analyzed, miR-21-5p silencing produced a significant increase (*p* < 0.05) in NFKB1 expression at early time points (Figure 7A). At 3 h, miR-21-5p silencing resulted in upregulation of NFKB1 expression by +67.4% compared with the IF-NC control. This effect remained significant at 6 h, but was reduced (+36.2%). The impact of miR-146a-5p silencing on NFKB1 at 6 h showed a significant upregulation of +51.1% (*p* < 0.05).

MYC regulation: At 3 h post-infection, inhibition of miR-21-5p led to a +77.3% increase (*p* < 0.05) in MYC expression relative to the control condition. By 6 h, the impact of miR-21-5p silencing remained detectable but was considerably reduced, and the regulatory pattern reversed, after dual inhibition, resulting in a decrease of −41.3% (*p* < 0.05) in this gene’s expression level (Figure 7B).

TNF regulation: Following miR-21-5p silencing, TNF expression was significantly reduced by −39.2% (*p* < 0.05) compared with the control. In contrast, miR-146a-5p inhibition and the combined inhibition had no significant effect on TNF regulation (Figure 7C).

IL18 regulation: Results show that anti-miR-21-5p increased IL18 expression relative to the control, and this upregulation increased significantly to 97.5% (*p* < 0.05) at 6 h. In contrast, inhibition of miR-146a-5p produced a slight decrease in IL18 expression over time. No significant changes were observed after dual inhibition of miR-21-5p and miR-146a-5p (Figure 7D).

XBP1 regulation: At 6 h, dual silencing of miR-21-5p and miR-146a-5p led to a significant increase in XBP1 expression levels with 57.1% (*p* < 0.01), which clearly exceeded the effects of either inhibitor alone (Figure 7E). Furthermore, we observed an earlier modulation of the active spliced form sXBP1. At 3 h, inhibition of miR-21-5p showed a significant downregulation of −43.8% (*p* < 0.05) of sXBP1’s expression level relative to the control (Figure 7F).

However, HIF1A expression levels remained stable following silencing of miR-21-5p, miR-146a-5p, or their combination, with no statistically significant deviations observed at early infection time points (Supplementary Figure S1A). Similarly, IL-8 expression showed only minor modulation (Supplementary Figure S1B). Moreover, we observed a progressive reduction in STAT3 expression following inhibition of miR-21-5p in both the mono- and dual-treatment groups (Supplementary Figure S1C).

Overall, miR-21-5p and miR-146a-5p inhibition produced distinct and time-dependent effects on the expression of the analyzed genes. miR-21-5p silencing significantly affected NFKB1, MYC, TNF, IL18, and sXBP1, whereas HIF1A, CXCL8, STAT3, XBP1, and the other genes analyzed remained unchanged under the conditions tested. miR-146a-5p silencing significantly affected only NFKB1, while no significant changes were observed for MYC, TNF, IL18, HIF1A, CXCL8, STAT3, XBP1, or sXBP1. Combined inhibition of miR-21-5p and miR-146a-5p significantly affected MYC and XBP1, whereas no significant changes were detected for NFKB1, TNF, IL18, HIF1A, CXCL8, STAT3, or sXBP1.

## 3. Discussion

The successful establishment of an in vitro infection model is crucial for studying the complex interactions between *Leishmania* parasites and host cells. To this end, we first demonstrated the presence of intracellular amastigotes in infected macrophages by MGG staining, where typical amastigote forms were observed by light microscopy. We observed a biphasic infection profile: an early infection phase (3–6 h) characterized by rapid colonization of the macrophage population (up to 69%), followed by a robust intracellular proliferation phase reaching 99% at 48 h. These observations corroborate our previous study by Lemaire et al. [13] with a slight difference in parasite load. These findings highlight the ability of *Leishmania* to invade macrophages and successfully replicate within them, suggesting a high level of parasite fitness within the phagolysosomal environment and indicating minimal host-mediated restriction of parasite growth in this model. Another group that infected THP1-like macrophages with *L. donovani* found a similar dynamic but with higher infection parameters [20], and this difference could be related to the parasite species, as noted in the study by Ramos et al. [21], who compared the in vitro infection of three *Leishmania* species and confirmed that the number of infected macrophages increased over time according to the parasite species. The dynamic nature of the infection process and its time-dependent characteristics may be attributed to various factors, namely: (i) the intracellular survival mechanisms of *Leishmania*, such as the ability to modulate host cell signaling pathways and interfere with immune responses, which likely play a crucial role in sustaining the infection; and (ii) the activation of immune cells and the production of cytokines and chemokines during infection, which can influence the infection rate and parasite load.

We acknowledge several limitations of this study. First, our infection model relies on the THP-1 monocytic cell line differentiated into macrophage-like cells, which, despite being widely used, does not fully recapitulate the phenotypic and functional heterogeneity of primary human monocyte-derived macrophages. Second, the miRNA-silencing and target-gene expression experiments were performed in technical replicates, from a limited number of biological replicates, which constrains the statistical power for detecting more subtle regulatory effects. Third, candidate target genes were identified through in silico prediction (miRNet) followed by functional perturbation at the transcript level; in the absence of direct biochemical assays (e.g., luciferase reporter or RNA-protein pull-down assays, which are out of the scope of this study), we cannot formally distinguish direct miRNA–target interactions from indirect, pathway-mediated effects. Finally, our analyses were restricted to the mRNA level, and no protein-level validation (e.g., Western blot or ELISA) was performed; because mRNA abundance, protein abundance, and secretion represent distinct levels of biological regulation that are not necessarily temporally synchronous, we considered transcript-level analysis an appropriate and informative first functional layer for assessing the consequences of miRNA perturbation during the early stages of infection. Future studies incorporating primary macrophages, larger biological replicate numbers, direct target-validation assays, and protein-level readouts will be needed to further substantiate and extend these findings.

The most remarkable aspect of our study is the identification, by qPCR, of differentially expressed miRs during *L. major* infection. MiRs are small non-coding RNA molecules that play crucial roles in post-transcriptional gene regulation, making them key players in various biological processes, including immune responses. In this context, it is important to note that the selection of a suitable housekeeping gene for normalizing miR expression is critical for ensuring accurate and reliable results. Small RNA molecules are commonly used as reference genes because of their size, stability, and abundant expression [22]. In our study, we evaluated the stability of three candidate housekeeping genes selected on the basis of previous literature reports: U6, U44, and the exogenous RNA spike-in UniSp6. Through comprehensive analysis using the multiple algorithms grouped in the online tool RefFinder, we determined that U6 and UniSp6 exhibited the best stability for normalizing miR expression. U6 is widely used as a reference gene in miR studies, and UniSp6, as an exogenous plate calibrator control, provides an additional level of assurance for the comparability of our results across different runs.

The differential expression pattern of these miRs provides valuable insights into the complex interplay between the host immune system and the invading parasite. Our results show that miR-21-5p and miR-146a-5p were rapidly induced upon activation of human macrophages (at 3 h post-infection), and their significant upregulation was well maintained up to 24 h post-infection, suggesting an important role as immune regulators in the early stages of infection. However, at 48 h post-infection, the expression of both miRs was significantly downregulated compared with the earlier time points. This observation suggests a potential regulatory mechanism whereby the expression of miR-21-5p and miR-146a-5p is controlled during the later stages of infection. Understanding the mechanisms underlying this dynamic expression pattern of these miRs will provide crucial insights into the host’s ability to mount an effective defense against *L. major*.

Our data demonstrate highly efficient and specific silencing of each miR by its specific anti-miR-LNA oligonucleotide, particularly during the critical early stages of infection. This robust and stable inhibition allows us to understand the immediate response of their predicted target genes in the first few hours of parasite entry. Furthermore, our results from the dual anti-miR silencing setup are encouraging. To our knowledge, this is the first study to successfully co-inhibit distinct, non-clustered miRs in a *L. major*-macrophage model and maintain independent 100% efficacy for both antimiRs. While Fernandes et al. [23] previously co-inhibited miRs belonging to the same cluster (miR-372/373/520d) in a *L. infantum* macrophage model [23], our approach targets two different miRs and provides a reliable framework for analyzing their cumulative effects on infection.

The decrease in miR-146a-5p inhibition efficiency over time led us to hypothesize a direct competition between the concentration of transfected anti-miR and new miR biogenesis. We suggest that, as the infection progresses, the expression of new miR-146a-5p molecules eventually outnumbers the remaining anti-miR. This may explain the observed drop in inhibition of this miR at later time points. For this reason, Lemaire et al. [13] used a double-transfection protocol to maintain high knockdown efficiency throughout the experimental kinetics [13,14].

Silencing of miR-146a and miR-21 was associated with enhanced inflammatory signaling at early time points, as evidenced by increased expression of NFKB1 and IL18. Given the established roles of miR-146a and miR-21 as negative regulators of innate immune responses, their downregulation may relieve inhibitory control over pro-inflammatory pathways, thereby promoting macrophage activation. Interestingly, at 3 h post-infection, miR-21 silencing was associated with increased MYC expression but reduced TNF and sXBP1 expression. These findings suggest that miR-21 plays a complex, context-dependent role during the early stages of infection, influencing cellular activation, inflammation, and ER stress-associated signaling.

## 4. Materials and Methods

### 4.1. Cell Culture and Differentiation

The human monocyte cell line THP-1 (ATCC TIB-202) was used in this study. Cells were cultured and maintained in RPMI 1640 medium (Sigma-Aldrich, Saint-Quentin Fallavier, France) supplemented with 2 mM L-glutamine, 100 U/mL penicillin, 100 U/mL streptomycin, and 10% inactivated Fetal Bovine Serum (FBS) (complete medium). The cells were incubated at 37 °C with 5% CO_2_. To differentiate THP-1 cells into macrophage-like cells, a concentration of 20 ng/mL of Phorbol Myristate Acetate (PMA, Sigma-Aldrich, Saint Louis, MO, USA) was added. A total of 0.5 × 10^6^ PMA-treated cells were then plated into 24-well plates in 1 mL of complete medium and incubated for 24 h under the same conditions. Following PMA treatment, the medium was replaced with fresh medium, and the differentiated cells were incubated for an additional 24 h before infection.

### 4.2. Parasite Culture and Metacyclic Promastigotes Purification

The parasite *L. major* MHOM/TN/94/GLC 94 [24] was maintained in RPMI 1640 medium supplemented with 2 mM L-glutamine and antibiotics (100 U/mL penicillin, 100 U/mL streptomycin) and 10% FBS (Hyclone, Logan, UT, USA), and incubated at 26 °C. Once the stationary phase was reached (day 6/7), the parasites were collected and resuspended at 1 × 10^8^ parasites/mL in Phosphate-Buffered Saline (PBS) and the metacyclic promastigotes were purified by negative selection using peanut agglutinin (PNA). The suspension was incubated for 30 min at room temperature before being centrifuged at 150g for 6 min to separate PNA− and PNA+ fractions as previously described [25]. Cell infections were conducted using purified metacyclic promastigotes. This ensured the use of homogeneous parasite forms, enabling consistency across experimental replicates.

### 4.3. In Vitro Infection

Macrophage-like THP-1 cells were infected at a ratio of 10 parasites per cell (10:1) and incubated for 3 h, 6 h, 24 h, and 48 h. As a positive control, cells were stimulated with 1 µg/mL of lipopolysaccharide (LPS). Non-infected (NI) and non-treated cells for each time point were maintained only in complete medium, serving as negative controls for the experiments. All conditions were prepared in triplicate, and the plates were incubated at 37 °C in a humid atmosphere with 5% CO_2_.

### 4.4. Parasitic Load and Infectivity

To evaluate parasite load, macrophage-like THP-1 cells were plated on sterile glass coverslips placed in 24-well culture plates, infected simultaneously with a parasite-to-cell ratio of 10:1, and incubated for different time points, as reported above. After incubation, the slides were washed 3 times with PBS. Cells were fixed with absolute methanol for 30 min and stained with May–Grünwald Giemsa (MGG). At least 100 macrophages were randomly selected at each time point post-infection and were counted under a light microscope to determine the infection percentage (percentage of infected macrophages out of total cells), as well as the parasitic load (average number of intracellular amastigotes per macrophage).

### 4.5. Total RNA Extraction and Quality Assessment

To enable the simultaneous profiling of mRNA and miR from each single sample, total RNA was extracted using the miRNeasy Mini Kit (Qiagen, Hilden, Germany). Cells were harvested and immediately lysed with 700 µL of QIAzol^®^ Lysis Reagent (Qiagen, Hilden, Germany), and the RNA extraction proceeded following the manufacturer’s instructions. RNA concentration and quality were determined using a NanoDrop micro-spectrophotometer and a Qubit fluorometer 3 (Thermofisher, Carlsbad, CA, USA).

### 4.6. RT and qPCR for mRNA

Total RNA was reverse-transcribed using the High-Capacity cDNA Reverse Transcription Kit (Applied Biosystems, Vilnius, Lithuania) according to the manufacturer’s instructions. qPCR was performed using SYBR Green PCR Master Mix (Applied Biosystems) with different primers to amplify selected genes: NFKB1, HIF1A, MYC, STAT3, TNF, CXCL8, IL18, XBP1, and sXBP1, with GAPDH used as the housekeeping gene (Supplementary Table S2). All qPCR reactions were performed on a QuantStudio 3 instrument (Applied Biosystems). The amplification was conducted with the following conditions: 95 °C for 10 min, 40 cycles at 95 °C for 15 s, and 60 °C for 60s. A non-template control was included for each primer pair as a negative control. The Ct values for each condition were normalized to those of GAPDH, and the relative expression levels were calculated using the 2^−ΔΔCt^ method. Fold-change values greater than 1.5 were considered up-regulated, and those with fold-change values lower than 0.5 were considered down-regulated.

### 4.7. Evidence-Based miRNA Candidate Selection

To identify the most biologically relevant miRs involved in the host response to *Leishmania* infection, we implemented a rigorous three-stage prioritization workflow. First, a comprehensive survey of the peer-reviewed literature was conducted to identify miRs with experimentally validated differential expression across both human and murine leishmaniasis models. This evidence-based list was subsequently cross-referenced with high-throughput in vitro screening data previously established in our laboratory, which identified 64 significantly dysregulated miRs in *L. major*-infected macrophages [13,14]. These datasets were then subjected to in silico functional filtering and network topology analysis. Potential target genes were predicted using miRNet, and priority was assigned to miRNAs demonstrating high degree centrality, particularly those targeting critical immunomodulatory nodes.

### 4.8. RT and qPCR for miR

The cDNA synthesis for miRs was performed using the miRCURY LNA RT kit (Qiagen, Hilden, Germany), starting from 10 ng/µL of total RNA in a reaction volume of 10 µL, according to the manufacturer’s instructions. A volume of 0.5 µL of UniSp6 spike-in included in the kit was added to the RT reaction mix as an RT positive control and as a plate calibrator among different qPCR runs. The RT reaction was performed at 40 °C for 60 min, then was inactivated at 95 °C for 5 min. qPCR was performed using the miRCURY LNA^®^ Probe^®^ PCR kit (Qiagen, Hilden, Germany) with primers (Qiagen, Hilden, Germany) and minor changes such as 1:20 diluted cDNA. To reduce run-to-run variation, each primer set was used in duplicate within the same run for every replicate obtained from the extraction. Additionally, a non-template control was included for each primer pair reaction as a negative control. The PCR protocol started with an initial incubation step of 2 min at 95 °C to activate the QuantiNova DNA Polymerase, followed by a two-step cycling (95 °C for 5 s and 56 °C for 30 s) for 45 cycles. All qPCR reactions were performed in a RotorGene Q instrument (Qiagen, Hilden, Germany). The relative expression levels were calculated using the 2^−ΔΔCt^ method and a cut-off of 1.2 was chosen to identify differentially expressed miRs [13,14]. An expression significantly higher than 1.2 is defined as upregulated, and an expression significantly lower than 0.8 is defined as downregulated.

### 4.9. Exogenous and Endogenous miRNA Controls for miRNA qPCR Normalization

To ensure that miR quantification was not affected by the technical variability that may be introduced at different analysis steps, the synthetic, non-human spike-in RNA “UniSp6” was added to all samples as an exogenous control during the preparation of the RT mix to allow for normalization of sample-to-sample variation and to monitor reverse transcription efficiency. Moreover, SNORD6 (U6) and SNORD44 (U44) were used as endogenous housekeeping genes to normalize miR expression. These reference gene candidates were chosen through a literature review covering studies on miR normalization in the context of *Leishmania* infection.

The stability of each candidate reference gene for miR was evaluated using the online-based tool RefFinder (http://blooge.cn/RefFinder/ (accessed on 25 February 2025)) [26,27]. It combines four of the major computational programs currently available, including BestKeeper, comparative DeltaCt, NormFinder, and GeNorm. These tools use different algorithms to identify the most stably expressed gene within a given sample set. To assess the stability of each candidate, we used their Ct values across all experimental conditions (NI, IF, LPS) and over time (kinetics) as input data. The lowest value output calculated by the software identifies the most stably expressed housekeeping gene.

### 4.10. Macrophage Transfection with miR Inhibitor

To investigate the regulatory roles of miR-21-5p and miR-146a-5p on predicted target-gene expression during *L. major* infection, macrophage-like THP-1 cells were transfected with specific miR inhibitors. Briefly, cells were transfected with either the miR-21-5p inhibitor, the miR-146a-5p inhibitor, or a co-transfection of both inhibitors. A non-targeting scrambled sequence (Qiagen) served as negative control. The miR-21-5p and miR-146a-5p inhibitors and the scrambled negative control are pre-designed LNA-enhanced miRNA inhibitors (Qiagen); their sequences and catalogue information are provided in Supplementary Table S3. All inhibitors were used at a final concentration of 50 nM [28]. According to the manufacturer’s protocol, macrophage-like cells were transfected using the HiPerFect Transfection Reagent (Qiagen, Hilden, Germany) for 6 h. After 6 h of incubation with the transfection complexes, 400 µL of complete culture medium supplemented with serum and antibiotics was added, and cells were incubated overnight to allow for optimal miR silencing before *L. major* infection, following the protocol described above. The inhibition percentage of predicted target genes after miR silencing was calculated relative to the scrambled IF-NC (negative control) baseline for each miR at the respective time point.

### 4.11. Prediction of miR Target Genes and Functional Analysis

To predict target genes of selected miRs, we used the bioinformatics tool miRNet V2.0 (https://www.mirnet.ca/ (accessed on 9 June 2026)) [29]. This online tool integrates multiple experimentally validated databases and combines algorithms to provide comprehensive information and visualization of miR–target interactions. The differentially expressed miRs were used as input in miRNet, and the predicted target genes were retrieved. To gain insights into the biological processes involved, a functional enrichment analysis was performed using the Kyoto Encyclopedia of Genes and Genomes (KEGG) pathway databases available on miRNet. We selected significant pathways (FDR < 0.05) and identified several target genes from pathways closely associated with the innate immune response during leishmaniasis. Gene selection was also guided by earlier studies identifying these targets as key mediators of the inflammatory response and regulators of *Leishmania* replication, thus ensuring their relevance and significance in the context of our study.

### 4.12. Statistical Analysis

The expression analyses of miR-21-5p, miR-146a-5p, miR-30a-3p, and miR-101-3p were performed using three independent biological replicates. The miRNA silencing experiments and subsequent target-gene expression analyses were performed in technical replicate using two independent biological replicates. Statistical analysis was performed using Graph Pad Prism 6.0 software (GraphPad Software, San Diego, CA, USA). Results are expressed as means ± Standard Error of Mean (SEM) of independent experiments. The data were evaluated by analysis of variance (ANOVA; one-way or two-way) for multiple-group comparisons and corrected by the Tukey test. *p* values < 0.05 (*), <0.01 (**), or <0.001 (***) were considered statistically significant.

## Data Availability

The original contributions presented in this study are included in the article. Further inquiries can be directed to the corresponding authors.

## Supplementary Materials

The following supporting information can be downloaded at https://www.mdpi.com/article/10.3390/ijms27198893/s1. Refs. [30,31,32] cited in Supplementary Materials.
