# Supplementary material for: microRNA and Regulatory Networks of Human Macrophage During Leishmania major In Vitro Infection
Source: Int J Mol Sci. 2026 Oct 7;27(19):8893. doi: 10.3390/ijms27198893 (PMC13666224; doi:10.3390/ijms27198893)
Supplement: Supplementary file 1 [file ijms-27-08893-s001.zip › Supplementary_Material_Habouria_et_al_2026_IJMS_Revised_Final.pdf]

Supplementary Materials

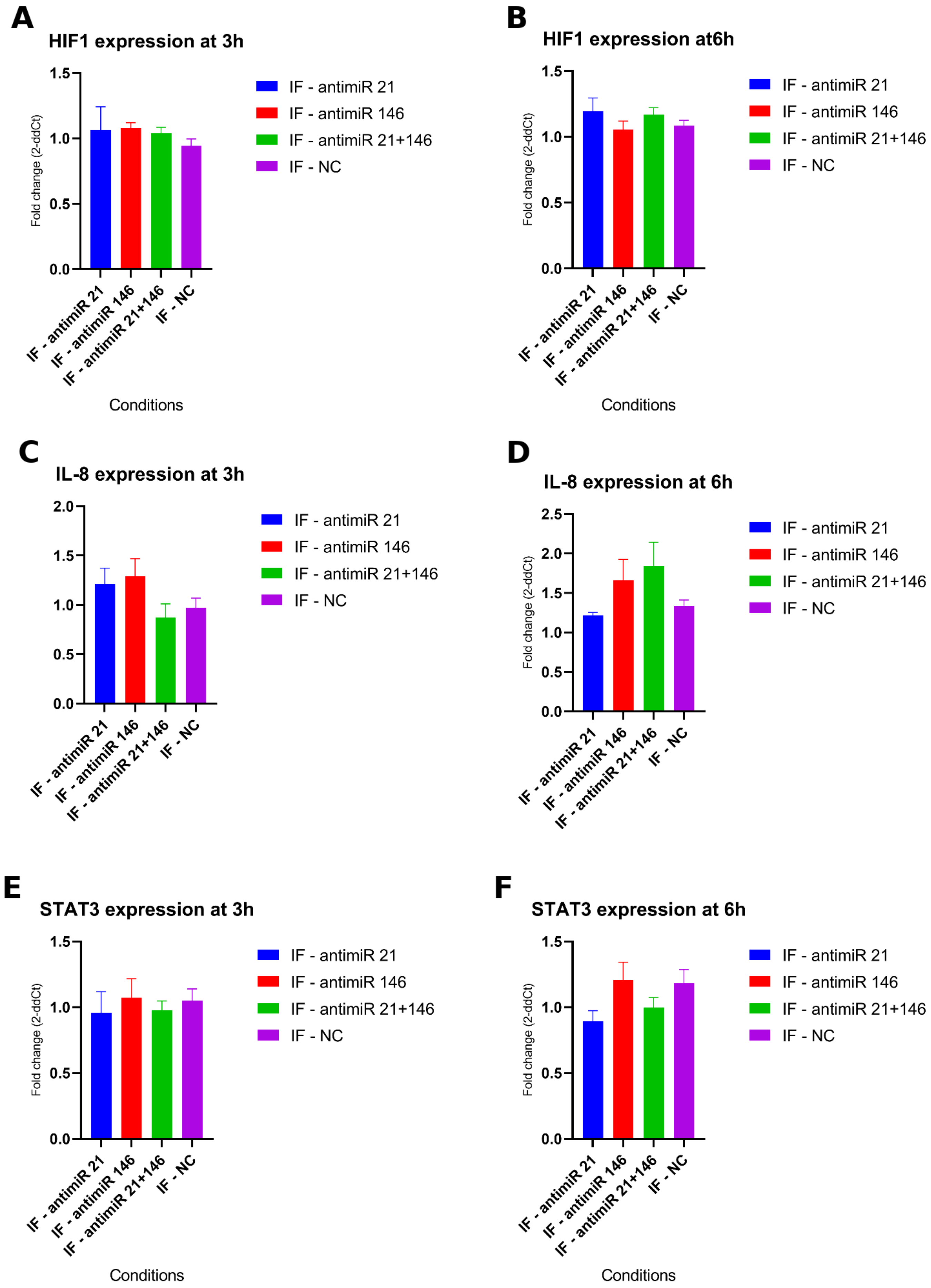
A
HIF1 expression at 3h
Fold change (2-ddCt)
1.5
1.0
0.5
0.0
IF - antimiR 21
IF - antimiR 146
IF - antimiR 21+146
IF - NC
Conditions
B
HIF1 expression at6h
Fold change (2-ddCt)
1.5
1.0
0.5
0.0
IF - antimiR 21
IF - antimiR 146
IF - antimiR 21+146
IF - NC
Conditions
C
IL-8 expression at 3h
2.0
1.5
1.0
0.5
0.0
IF - antimiR 21
IF - antimiR 146
IF - antimiR 21+146
IF - NC
D
IL-8 expression at 6h
Fold change (2-ddCt)
2.5
2.0
1.5
1.0
0.5
0.0
IF - antimiR 21
IF - antimiR 146
IF - antimiR 21+146
IF - NC
E
STAT3 expression at 3h
Fold change (2-ddCt)
1.5
1.0
0.5
0.0
IF - antimiR 21
IF - antimiR 146
IF - antimiR 21+146
IF - NC
Conditions
F
STAT3 expression at 6h
Fold change (2-ddCt)
1.5
1.0
0.5
0.0
IF - antimiR 21
IF - antimiR 146
IF - antimiR 21+146
IF - NC
Conditions

Supplementary Figure S1. HIF1A, IL-8, and STAT3 expression following miR-21-5p, miR-146a-5p, or dual silencing at early time points post-infection. (A,B) HIF1A expression at 3 h and 6 h post-infection remained stable across all silencing conditions (IF - antimiR21, IF - antimiR146, IF - antimiR21+146) relative to the scrambled negative control (IF - NC). (C,D) IL-8 expression at 3 h and 6 h post-infection showed only minor, non-significant modulation across conditions. (E,F) STAT3 expression at 3 h and 6 h post-infection showed a modest, progressive reduction following miR-21-5p inhibition, in both the mono- and dual-treatment groups. Data are shown as fold change ($2^{-\Delta\Delta Ct}$) relative to the scrambled IF-NC baseline; error bars represent SEM.

Supplementary Table S2. List of qPCR primers used for mRNA and miR targets.

Target Gene	Accession Number	Primers (5'–3')	Reference
MYC	NM_002467	FW: CGTCCTCGGATTCTCTGCTC<br>RV: TTGTTCCTCCTCAGAGTCGC	[30]
NFKB1	NM_003998	FW: TAATGCCTTCCGGCTGAGTC<br>RV: TGCTTCGGTGTAGCCCATTT	—
HIF1A	NM_181054	FW: GCTTTAACTTTGCTGGCCCC<br>RV: CTGTGTCGTTGCTGCCAAAA	—
STAT3	NM_001384989	FW: CTCCTTGCCAGTTGTGGTGA<br>RV: TCTTGGGATTGTTGGTCAGC	—
IL10	NM_000572	FW: CATCGATTTCTTCCCTGTGAA<br>RV: TCTTGGAGCTTATTAAAGGCATTC	[31]
IL18	NM_001562	FW: TGACTGTAGAGATAATGCACCCC<br>RV: AGTTACAGCCATACCTCTAGGC	[28]
XBP1	NM_001079539	FW: CCGCAGCACTCAGACTACG<br>RV: TGTCCAGAATGCCCAACAGG	[32]
sXBP1	NM_001079539	FW: CTGAGTCCGCAGCAGGT<br>RV: TGTCCAGAATGCCCAACAGG	[28]
CXCL8	NM_001354840	FW: CACTGCGCCAACACAGAAATT<br>RV: ACTTCTCCACAACCCTCTGCA	—
TNF	NM_000594	FW: TCTCTAATCAGCCCTCTGGCC<br>RV: TGGGCTACAGGCTTGTCACTC	—

MYC, MYC proto-oncogene, bHLH transcription factor; NFKB1, nuclear factor kappa B subunit 1; HIF1A, hypoxia inducible factor 1 subunit alpha; STAT3, signal transducer and activator of transcription 3; IL10, interleukin 10; IL18, interleukin 18; XBP1/sXBP1, X-box binding protein 1 (unspliced/spliced); CXCL8, C-X-C motif chemokine ligand 8; TNF, tumor necrosis factor.

Supplementary Table S3. List of miR inhibitors and scrambled sequence used for miR silencing.

miR inhibitor	Sequence (5'–3')	Reference
Anti-miR-21-5p	CAACATCAGTCTGATAAGCT	has-miR-21-5p miRCURY LNA miRNA Inhibitor (YI04100689 , Qiagen, https://geneglobe.qiagen.com/us/product-groups/mircury-lna-mirna-inhibitors/YI04100689)
Anti-miR-146a-5p	ACCCATGGAATTCAGTTCTC	has-miR-146a-5p miRCURY LNA miRNA Inhibitor (YI04100676, Qiagen, https://geneglobe.qiagen.com/us/product-groups/mircury-lna-mirna-inhibitors/YI04100676)
Scrambled- sequence	TAACACGTCTATACGCCCA	miRCURY LNA miRNA Inhibitor Control (Qiagen)
